# Use of a Piezoelectric Bender Element for the Determination of Initial and Final Setting Times of Metakaolin Geopolymer Pastes, with Applications to Laterite Soils

**DOI:** 10.3390/s22031267

**Published:** 2022-02-07

**Authors:** Janjit Iamchaturapatr, Keeratikan Piriyakul, Aruz Petcherdchoo

**Affiliations:** 1 Center of Excellence in Structural Dynamics and Urban Management, Department of Civil and Environmental Engineering Technology, College of Industrial Technology, King Mongkut’s University of Technology North Bangkok, 1518 Pracharat 1 Road, Wongsawang, Bangsue, Bangkok 10800, Thailand; janjit.i@cit.kmutnb.ac.th; 2 Department of Civil Engineering, Faculty of Engineering, King Mongkut’s University of Technology North Bangkok, 1518 Pracharat 1 Road, Wongsawang, Bangsue, Bangkok 10800, Thailand; aruz.p@eng.kmutnb.ac.th

**Keywords:** metakaolin, geopolymer, shear wave velocity, setting time, laterite soil

## Abstract

This study proposes the use of a non-destructive testing technique, based on piezoelectric bender element tests, to determine the initial and final setting times of metakaolin geopolymer pastes. (1) Background: Metakaolin geopolymer is a new eco-friendly building material that develops strength rapidly and is high in compressive strength. (2) Methods: The initial and the final setting times were investigated via bender element and Vicat needle tests. Metakaolin powder was prepared by treating kaolin at 0, 200, 800, 1000, and 1200 °C. All metakaolin powder samples were then mixed with geopolymer solution at different mixing ratios of 0.8:1.0, 1.0:1.0, 1.2:1.0, and 1.5:1.0. The geopolymer solution was prepared by adding 10 normal concentrations of sodium hydroxide (10 N NaOH) to sodium silicate (Na_2_SiO_3_) at various solution ratios of 1.0:1.0, 1.0:1.2, 1.0:1.5, 1.0:2.0, 1.2:1.0, 1.5:1.0 and 2.0:1.0. (3) Results: The optimum temperature for treating metakaolin is established at 1000 °C, with a mixing ratio between the metakaolin powder and the geopolymer solution of 1.0:1.0, as well as a solution ratio between NaOH and Na_2_SiO_3_ of 2.0:1.0. (4) Conclusions: The use of piezoelectric bender elements to determine the initial and final setting times of metakaolin geopolymer pastes is a useful method by which to detect geopolymerization by shear wave velocity in a real-time manner. Moreover, the penetration of the Vicat apparatus can confirm the setting times at specific intervals. The relationships between the shear wave velocity and the Vicat penetration appear to be linear, with an initial setting time of 168 m/s and a final setting time of 187 m/s. Finally, the optimum metakaolin geopolymer pastes are applied to improve laterite soils, as measured by CBR tests.

## 1. Introduction

Various kinds of techniques are available in the marketplace for determining the properties of materials. The criterion by which to select a suitable technique depends on the properties to be determined. The bender element test is considered to be one of the outstanding non-destructive tests for numerous reasons, such as its capacity for real-time monitoring [1]. In combination with piezoelectric sensors, the bender element can effectively determine the shear wave velocity and the initial shear modulus of materials in studies [2,3,4,5,6]. As an example, the bender element test was applied to studying soil characteristics, e.g., thawing and stress restoration in artificial frozen sandy soils [7], transition evaluation of fine sand from capillarity to cementation [8], expansion-induced crack propagation monitoring in rocks [9], response monitoring of transitional mixtures retaining the memory of in-situ overburden pressure [10], etc. For concrete materials, various studies can also be carried out using the bender element, e.g., the characterization of recycled concrete aggregate [11], evaluation of damage induced by alkali-silica reaction [12], etc.

Other than the aforementioned applications in terms of soil and concrete, the piezoelectric bender element can be used to determine the properties of those cement materials that are popular in construction due to the fact that they are both common in the marketplace and cost-effective. One of their essential properties to be determined is their setting time. As an example, Zhu et al. [13] utilized the bender element technique to determine the cement mortar setting times, using shear wave velocity evaluation curves. In their study, specimen geometry and the bender element were set to obtain shear wave velocity evaluation curves at varying times, as well as for different water-to-cement ratios and chemical admixtures. Accordingly, derivative methods were proposed by which to obtain the initial and the final setting times. It was found that the final setting time was correlated with the first-order derivative curves, while the initial setting time corresponded to the second-order derivative curves. Liu et al. [14] used piezoceramic bender elements in combination with ultrasonic shear waves to monitor the setting process of mortar and concrete. It was observed that the mortar, which was tested using different water-to-cement ratios, showed a clear relationship between shear wave velocity and penetration resistance. Moreover, the shear wave was more reliable than the P-wave for monitoring the setting process of cementitious materials. Reinhardt and Grosse [15] developed a device producing ultrasound waves, in order to continuously monitor the setting and hardening of cementitious materials. It was found that the beginning of setting could be determined from the relationship between velocity and the age of mortar, using a mathematical procedure. However, the final setting still relied on empirical experience. This method was considered to be applicable for concrete, gypsum, lime, starch, and other stiffening materials. It could also be used for quality control in the production of admixtures, as well as new binders, and for control of the consistency of concrete production.

In spite of its aforementioned advantages, however, cement production requires high energy input, leading to environmental concerns [16,17]. Hence, a new alternative building material is required for civil engineering works. As such, metakaolin, which is basically made from kaolin (a type of clay) treated using a high calcination temperature [18,19], is considered to be one of the possible replacement materials. In spite of the calcination process necessary in metakaolin production, Sullivan et al. [20], who reviewed the study by Selmani et al. [21], stated that the manufacture of metakaolin involved much lower calcining temperatures and emitted less carbon dioxide than Portland cement. Therefore, metakaolin is gaining popularity in various construction applications, as mentioned in other studies [22,23]. As one example of its applications, Attanasio et al. [24] used metakaolin, fly ash and furnace slag to produce alkali-activated mortars, in order to study the effect of binder compositions by focusing on workability and compressive strength. It was revealed that such a cement-free binder had the potential for using it as masonry mortar. Nas and Kurbetci [25] investigated the durability properties of concrete containing metakaolin. It was found that the optimum metakaolin substitution ratio was 20%. Moreover, the use of metakaolin in concrete improved its durability properties, such as sorptivity, permeability, and durability.

Another application of metakaolin is its use in producing geopolymer, which was considered as one of the possible products due to its advantages in economic and environmental terms, as well as its rapid strength development, etc. [26]. It is observed that although the geopolymer can fundamentally be made using different materials, such as fly ash, limestone powder, rice husk ash, water treatment plant sludge, blast-furnace waste, silica fume, etc., much of the research still focuses on metakaolin geopolymer due to its availability in many places. As an example, Elimbi et al. [27] produced geopolymer from a dehydroxylated form of natural kaolin with a calcination temperature of between 450 and 800 °C. Accordingly, it was reported that the most suitable temperature for kaolin calcination was around 700 °C. Alaskar et al. [28] stated that the metakaolin geopolymer could be activated by heating clay in a furnace chamber at 600, 700, and 800 °C for 1, 2, and 3 h, respectively, or at 900 and 1000 °C for 1 and 2 h, respectively. Moreover, the maximum compressive strength in terms of calcination was found at 700 °C for 1 h. Abadel et al. [29] studied the strength, microstructure, and embodied energy of geopolymer mixtures by varying the ratio of the alkali solids to metakaolin from 0.1 to 0.5, as well as varying the ratio of sodium silicate to NaOH from 0.2 to 1.0. They found that when manufacturing a metakaolin geopolymer with a strength of 90 MPa, the molar ratio of silica to alumina should be greater than 2.3. Moreover, the ratio of sodium oxide to alumina should be between 0.6 and 1.2, and the ratio of water to sodium oxide should not exceed 12. Palumbo et al. [30] used fiber Bragg grating sensors to characterize the early-age curing and shrinkage of metakaolin geopolymer. It was found that their method could accurately control the early-age phases of the metakaolin geopolymer. Apart from these studies, metakaolin geopolymer can further be applied to civil engineering works, such as a low-cost and self-bearing thermal insulating core for thermostructural sandwich panels [31], cultural heritage restoration [32], and as a potential binder for granulation zeolites [33,34]. From these applications, it can be observed that various research topics in metakaolin geopolymer are still ongoing.

According to the aforementioned review, it can be observed that bender element testing has been applied to various research areas, in particular, to the setting time of materials. However, there is no previous research that has applied the bender element test to determine the setting times of metakaolin geopolymer pastes. In order to bridge this gap, this study proposes to use the piezoelectric bender element to investigate the initial and final setting times of metakaolin geopolymer pastes. For the experiment, metakaolin powder was prepared by treating kaolin at various temperatures of between 0 and 1200 °C. The metakaolin powder was then mixed with geopolymer solution at various mixing ratios. In addition, the geopolymer solution was prepared by adding 10 normal sodium hydroxide (10 N NaOH) concentrations to sodium silicate (Na_2_SiO_3_) at various solution ratios. Finally, the results from the bender element test, in terms of the setting times of metakaolin geopolymer pastes, were compared to those from the Vicat needle test, whereby the optimum mixing and solution ratios could be established. Moreover, the obtained optimum ratio is used to produce metakaolin geopolymer pastes for mixing with laterite soil samples. After this, the California bearing ratio (CBR) test was used for comparing laterite soils mixed with metakaolin geopolymer to control laterite soils.

## 2. Materials and Methods

### 2.1. Metakaolin Geopolymer

**Figure 1:**
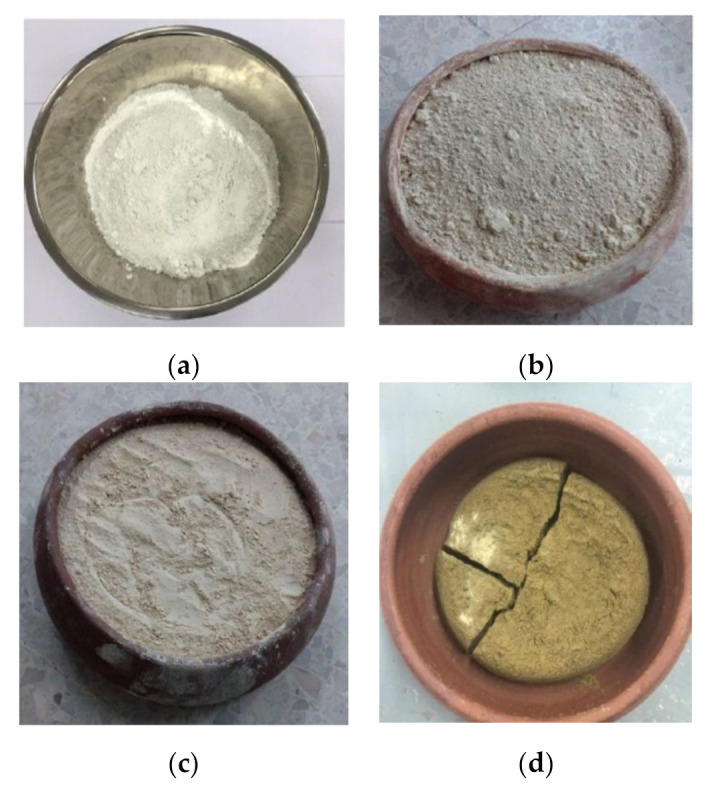
Kaolin after thermal treatment at various temperatures: (**a**) 200 °C; (**b**) 800 °C; (**c**) 1000 °C; (**d**) 1200 °C.

This study used kaolin with the chemical composition and basic engineering properties shown in Table 1 and Table 2, respectively. Here, the particles of kaolin appeared to be very fine and passed easily through a number 400 ASTM mesh (38 μm). For control samples, the kaolin was not thermally treated and was designated as 0 °C. For the metakaolin geopolymer paste samples, the kaolin was treated at different temperatures from 200 to 1200 °C to obtain metakaolin powder, as shown in Figure 1a–d, respectively. It was observed that the metakaolin that was treated at 1200 °C, as shown in Figure 1d, appears to be ceramic and cannot be mixed with geopolymer solution. Therefore, it was not considered throughout the study. After thermal treatment, the metakaolin powder that was treated at 200 to 1000 °C was then mixed with a geopolymer solution. The mixing ratios between the metakaolin powder and the geopolymer solution were selected as 0.8:1.0, 1.0:1.0, 1.2:1.0, and 1.5:1.0. The geopolymer solution was prepared by adding 10 normal concentrations of sodium hydroxide (10 N NaOH) to the sodium silicate (Na_2_SiO_3_) at various solution ratios of 1.0:1.0, 1.0:1.2, 1.0:1.5, 1.0:2.0, 1.2:1.0, 1.5:1.0, and 2.0:1.0.

### 2.2. Piezoelectric Transducers

**Figure 2:**
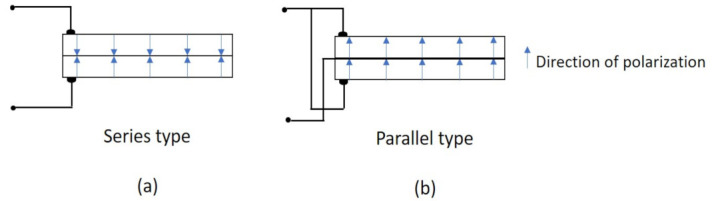
Piezoelectric ceramic transducers: (**a**) series type; (**b**) parallel type.

The piezoelectric ceramic transducer used here is a bimorph-type vibrator, fabricated by the Fuji ceramics corporation. Basically, this transducer is connected with two pieces of piezoelectric ceramic. When one piece is extended, another piece is compressed. Using this mechanism, the transducer can vibrate and send shear waves. For this transducer, there are two different connection methods, series and parallel types, as shown in Figure 2a,b, respectively. The series type can be used for the highly sensitive sensing of a faint signal, while the parallel type can be used as an actuator due to its large displacement at a low voltage. Regarding these two types, further explanations will follow later.

### 2.3. Shear Wave Velocity Measurement

**Figure 3:**
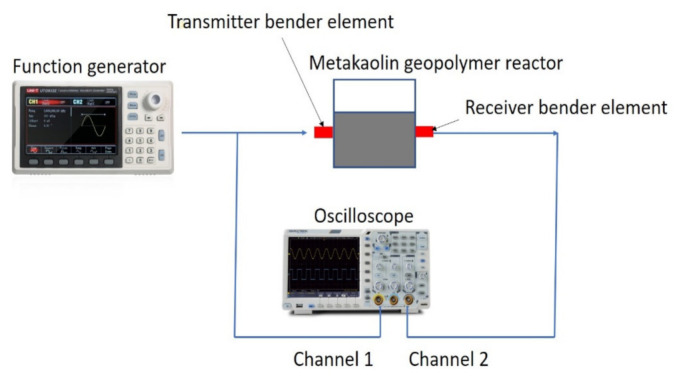
Bender element test setup.

Working according to the ASTM D8295–19 (the standard test method for the determination of shear wave velocity and initial shear modulus in soil specimens using bender elements) [35], the shear wave is generated here by a function generator with a sinusoidal waveform, with a frequency of 4–10 kHz and an amplitude of 20 V_p-p_. After generation, the shear wave is sent to the transmitter of the bender element, in combination with measuring the sending shear wave via channel 1 of the oscilloscope, as shown in Figure 3. For this sending transmitter, a parallel-type connection is used due to its large deformation and strong sending signal. After leaving the sending transmitter, the shear wave travels through the metakaolin geopolymer paste within the reactor (in a crystal-clear plastic container) as shown in Figure 3. At the receiving bender element on the opposite side, a series-type connection is used due to its high sensitivity. Moreover, the receiving shear wave is detected via channel 2 of the oscilloscope, as shown.

**Figure 4:**
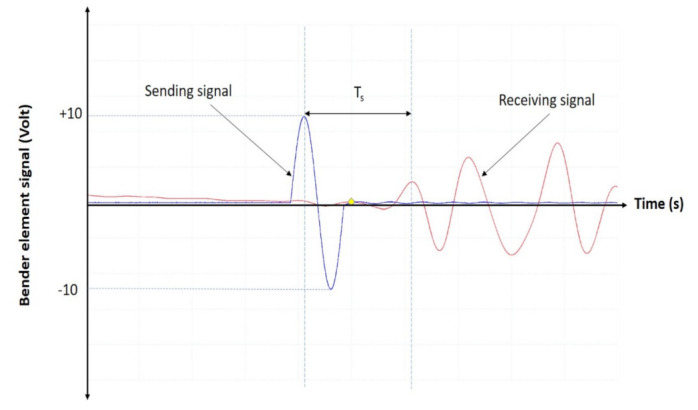
Example of sending and receiving shear waves.

Figure 4 shows an example of sending and receiving shear wave signals, and its offset time is known as shear wave travel time, *T_s_* (s). This offset time is used to calculate the shear wave velocity *v_s_* (m/s), as follows:

(1) $$v_{s}=\frac{\left(L\right)}{\left(T_{s}-T_{c}\right)}$$

where *L* is defined as the length of the metakaolin geopolymer specimen measured between two bender element transducers (m), whereas *T_c_* is defined as the time delay correction (s).

### 2.4. Vicat Needle

**Figure 5:**
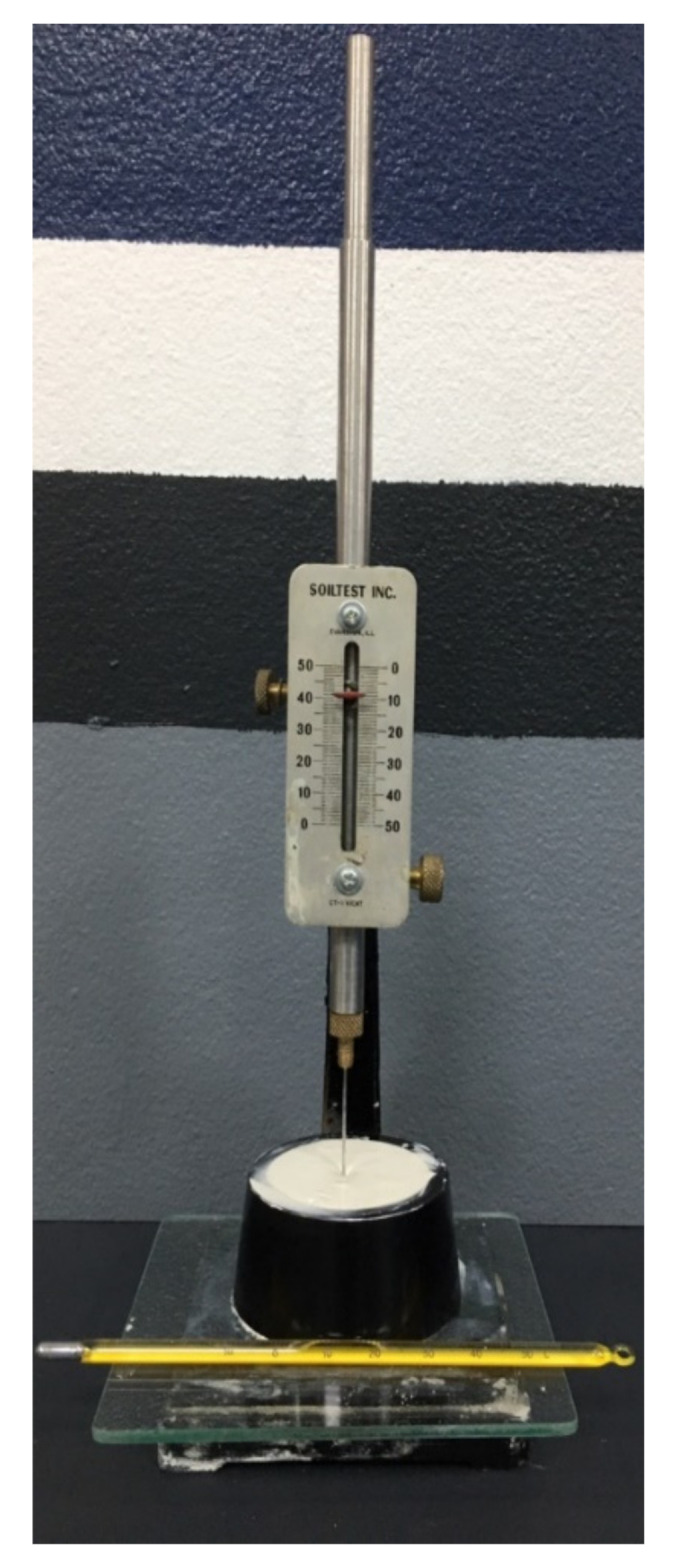
Vicat needle test apparatus.

According to ASTM C191-08 (standard test methods for measuring the setting time of hydraulic cement with a Vicat needle), the Vicat needle test apparatus [36] in Figure 5 can be used to determine both the initial and the final setting times of metakaolin geopolymer pastes. In this study, the initial setting time is judged as a Vicat penetration of 25 mm, while the final setting time is judged to be whenever there is no occurrence of Vicat penetration.

### 2.5. Laterite Soil

For this study, a set of laterite soil samples were taken from the Bo Phloi District, Kanchanaburi, Thailand. These were classified as sandy clay (CL) according to the unified system, or A-4 based on the AASHTO system. Table 3 shows the soil engineering properties of the laterite soil samples. These laterite soil samples were used in the CBR tests in accordance with ASTM D1883-99. For these tests, 3 different mixing methods were carried out, i.e., A, B, and C. For mixing method A, the laterite soil was blended together 5 separate times with a mixture of metakaolin and geopolymer solution, then each of these 5 mixtures was assembled layer by layer. For mixing method B, the laterite soil and the metakaolin were first mixed together, then the geopolymer solution was subsequently added. For mixing method C, both the metakaolin and the geopolymer solution were first mixed together, then the laterite soil was mixed in later. It is noteworthy that in these tests, the solution ratio between NaOH and Na_2_SiO_3_, as well as the mixing ratio between the metakaolin and the geopolymer solution, were selected based on the results of the bender element tests.

## 3. Results and Discussions

### 3.1. Effect of Thermal Treatment on Metakaolin

**Figure 6:**
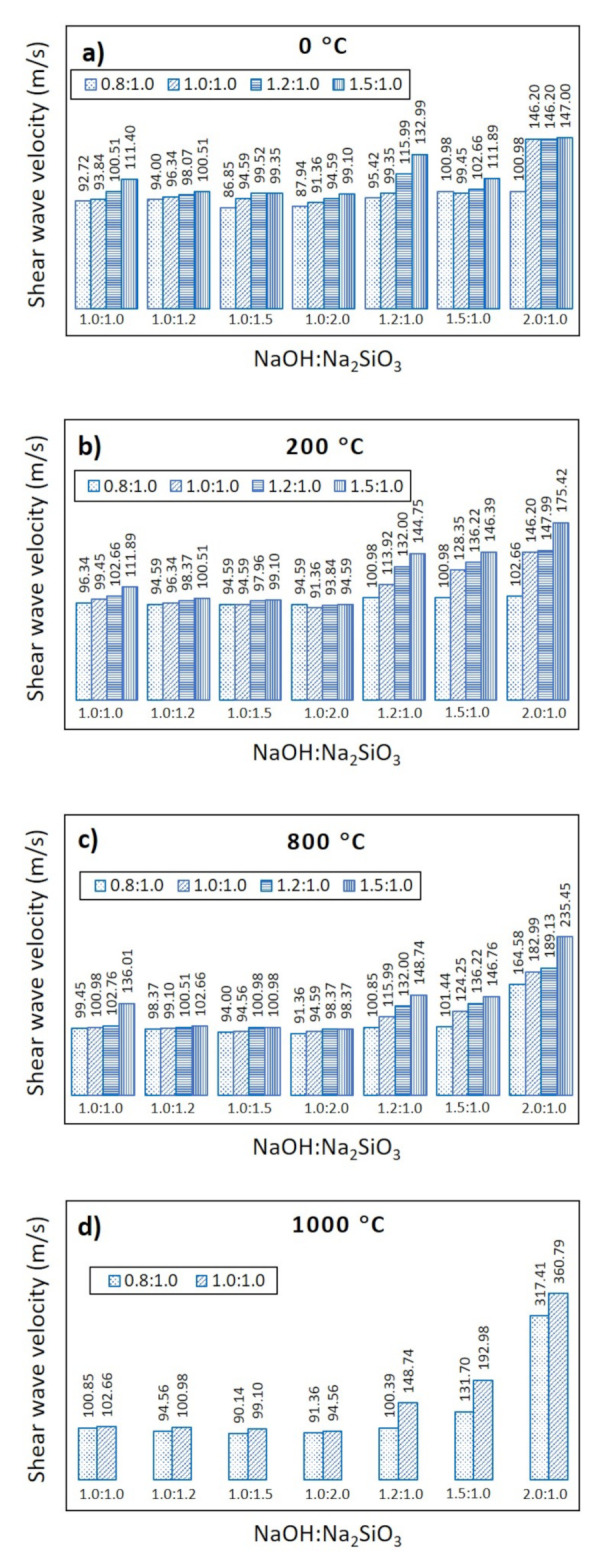
Shear wave velocity vs. NaOH:Na_2_SiO_3_, with various ratios of metakaolin powder and geopolymer solution and various metakaolin treatment temperatures: (**a**) 0 °C; (**b**) 200 °C; (**c**) 800 °C; (**d**) 1000 °C.

Figure 6 shows the relationship between the shear wave velocity, V_s_, through metakaolin geopolymer pastes measured for 180 min and the solution ratio of NaOH and Na_2_SiO_3_, measured at various mixing ratios of metakaolin powder and geopolymer solution. Figure 6a shows the scenario of the control sample with no thermal treatment, i.e., 0 °C. It was found that the maximum V_s_ is about 147 m/s when the solution ratio between NaOH and Na_2_SiO_3_ is equal to 2.0:1.0, as well as a mixing ratio between the metakaolin powder and the geopolymer solution equal to 1.0:1.0, 1.2:1.0, and 1.5:1.0. With the same solution ratio and the same mixing ratio, Figure 6b,c show that the maximum V_s_ is about 147–175 m/s for metakaolin treated at 200 °C, and about 183–235 m/s for metakaolin treated at 800 °C, respectively. Figure 6d shows the scenario of metakaolin when it is treated at 1000 °C. It can be seen that V_s_ reaches a maximum at 360 m/s, when the solution ratio is equal to 2.0:1.0 and the mixing ratio is equal to 1.0:1.0. However, it is clear that V_s_ at a solution ratio of 1.2:1.0 and 1.5:1.0 is not available when the temperature treatment is at 1000 °C, because geopolymerization was found to be so rapid that the setting time could not be measured. This is one of the significant limitations in utilizing the metakaolin geopolymer in real-life civil engineering work. Again, the metakaolin sample treated at 1200 °C was not considered as a variable throughout this study because the sample turned into a ceramic after thermal treatment, meaning that it was impossible to mix it with the geopolymer solution, as mentioned in Section 2.1.

**Figure 7:**
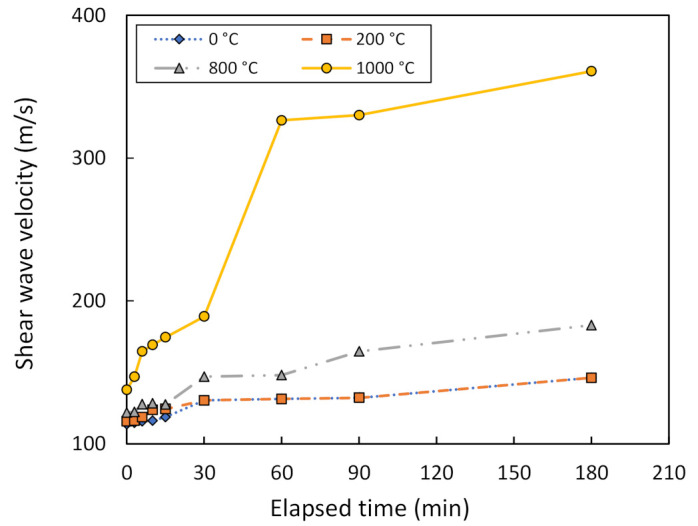
Shear wave velocity vs. the elapsed time of bender element testing, measured at different treatment temperatures.

Figure 7 shows the relationship between the shear wave velocity, V_s_, and the elapsed time of bender element testing on metakaolin geopolymer pastes at different treatment temperatures. For comparison purposes, the solution ratio between NaOH and Na_2_SiO_3_ was measured at 2.0:1.0, while the mixing ratio between the metakaolin powder and the geopolymer solution was measured at 1.0:1.0. It was observed that V_s_ increased with the passage of time. When using metakaolin treated at 0 and 200 °C, the V_s_ of both is identical and increases from about 115 m/s to 146 m/s after 180 min of testing, showing no significant geopolymerization in terms of V_s_. For metakaolin treatment at 800 °C, V_s_ increases from 122 to 183 m/s, or by 50%. However, for metakaolin treatment at 1000 °C, the V_s_ drastically increases from 138 to 361 m/s, or by 162%. Therefore, the metakaolin treatment temperature at 1000 °C is considered to be optimum in this study.

**Figure 8:**
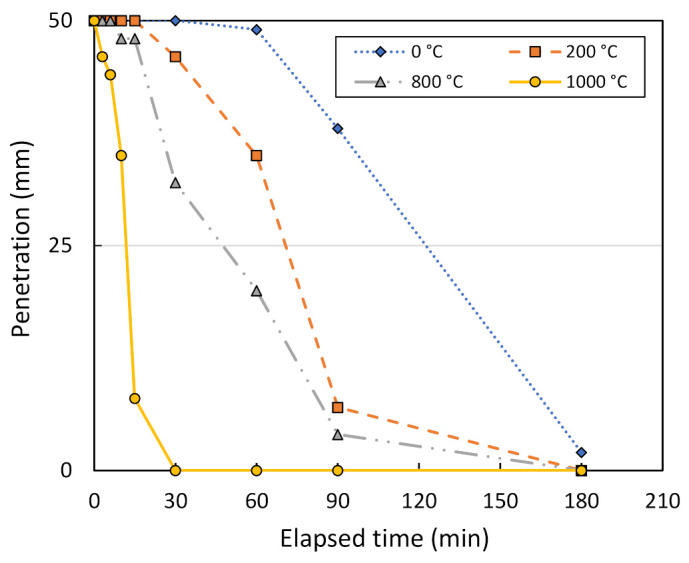
Penetration vs. the elapsed time of Vicat needle testing, measured at different treatment temperatures.

Figure 8 shows plots between Vicat penetration through metakaolin geopolymer pastes and the elapsed time, as the solution ratio and the mixing ratio were assumed to be 2.0:1.0 and 1.0:1.0, respectively. From the plot, it can be seen that the initial setting time for a 25-mm penetration of the Vicat apparatus was faster with a higher metakaolin treatment temperature. For the control sample designated as 0 °C, the initial setting time of metakaolin geopolymer pastes was equal to 123 min. Using the metakaolin treated at 200, 800, and 1000 °C, the initial setting time became faster at 71, 48, and 12 min, respectively. As measured until there was no penetration of the Vicat needle, the final setting time for metakaolin treated at 1000 °C was about 30 min, whereas that at other temperatures was about 180 min or more. This seems to agree with the test results of V_s_, as shown in Figure 7, where a temperature of 1000 °C was considered to be the optimum metakaolin treatment temperature.

### 3.2. Effect of the Ratio of NaOH: Na_2_SiO_3_ for Preparing the Geopolymer Solution

**Figure 9:**
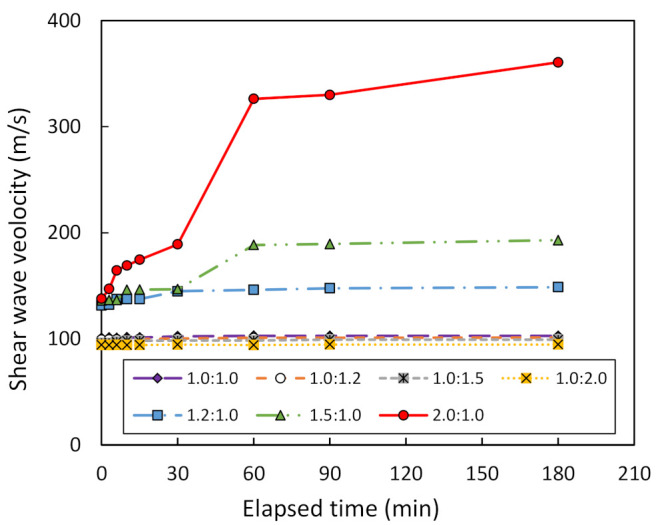
Shear wave velocity vs. elapsed time of bender element testing for various ratios of NaOH to Na_2_SiO_3_.

**Figure 10:**
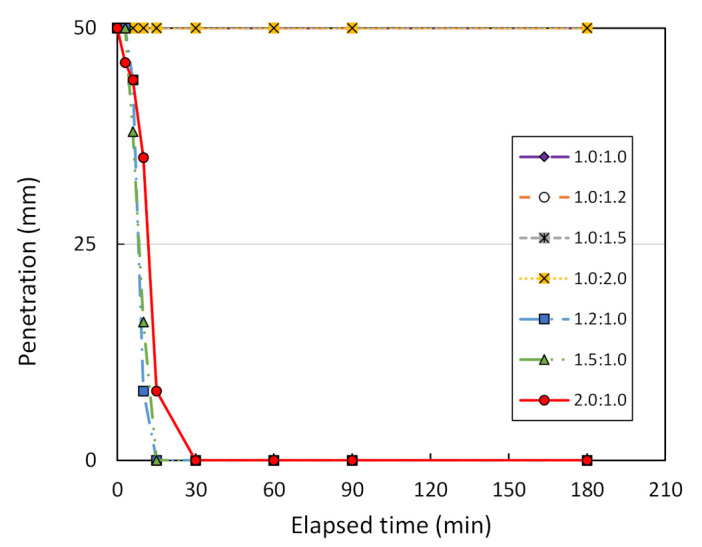
Penetration vs. elapsed time of Vicat needle testing for various ratios of NaOH to Na_2_SiO_3_.

The effect of varying the solution ratio between NaOH and Na_2_SiO_3_ on shear wave velocity, V_s_, and the Vicat penetration can be investigated. For comparison purposes, this study assumes the mixing ratio between metakaolin treated at 1000 °C and the geopolymer solution to be 1.0:1.0. Figure 9 plots the relationship between V_s_ and the elapsed time of bender element testing on metakaolin geopolymer pastes. It can be seen that V_s_ remains constant when the solution ratio is equal to 1.0:1.0, 1.0:1.2, 1.0:1.5 and 1.0:2.0. This means that there is no occurrence of geopolymerization. However, the geopolymerization occurs when the solution ratio is equal to 1.2:1.0, 1.5:1.0, and 2.0:1.0. Moreover, the V_s_ increases from 131 to 149 m/s and 136 to 193 m/s when the solution ratio is equal to 1.2:1.0 and 1.5:1.0, respectively. At a solution ratio of 2.0:1.0, V_s_ increases from 138 m/s to its maximum at 361 m/s. Hence, the optimum solution ratio between NaOH and Na_2_SiO_3_ is recommended here as being 2.0:1.0. Furthermore, Figure 10 presents the Vicat needle test results for metakaolin geopolymer pastes mixed at various solution ratios. It is clear that Vicat penetration is equal to 50 mm after 180-min testing for a solution ratio of 1.0:1.0, 1.0:1.2, 1.0:1.5, and 1.0:2.0. This shows that there is no setting of the metakaolin geopolymer pastes, meaning that there is no occurrence of geopolymerization. However, geopolymerization occurs only in the case of a solution ratio at 1.2:1.0, 1.5:1.0, and 2.0:1.0, since the initial setting time is equal to 8, 8, and 12 min, respectively. Moreover, the corresponding final setting time is equal to 15, 15, and 30 min, respectively.

### 3.3. Effect of Mixed Proportions between the Metakaolin and the Geopolymer Solution

**Figure 11:**
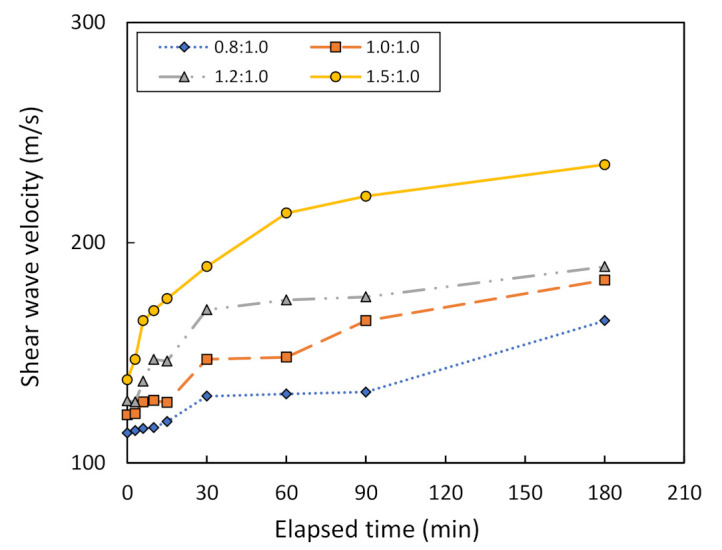
Shear wave velocity vs. the elapsed time of bender element testing for metakaolin treated at 800 °C, using different mixing ratios.

**Figure 12:**
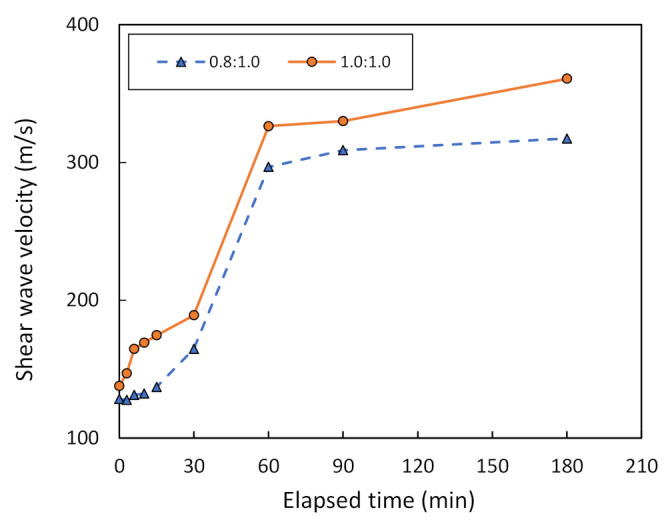
Shear wave velocity vs. the elapsed time of bender element testing for metakaolin treated at 1000 °C, using different mixing ratios.

The relationship between the shear wave velocity, V_s_, and the elapsed time of bender element testing, as well as between the penetration and the elapsed time of Vicat testing, can be used to determine the effect of varying the mixing ratio between the metakaolin powder and the geopolymer solution. For comparison purposes, this study assumes the solution ratio to be between NaOH and Na_2_SiO_3_ at 2.0:1.0 in combination with the metakaolin treated at 800 °C and 1000 °C. Figure 11 shows that V_s_ tends to increase with elapsed time in the case of the metakaolin treated at 800 °C. Moreover, the V_s_ at 180 min of bender element testing was equal to 165, 183, 189, and 235 m/s, when the mixing ratio is equal to 0.8:1.0, 1.0:1.0, 1.2:1.0, and 1.5:1.0, respectively. Hence, the mixing ratio of 1.5:1.0 is considered to be optimum in this study due to the maximum V_s_. In the same way, Figure 12 reports the relationship between V_s_ and the elapsed time of bender element testing on metakaolin treated at 1000 °C. Only the mixing ratio at 0.8:1.0 and 1.0:1.0 is measured, because the metakaolin mixed using the other ratios could not be tested due to rapid drying, as mentioned in Section 2.1. It can be seen that the V_s_ for a mixing ratio of 1.0:1.0 is higher than that of 0.8:1.0. For example, the V_s_ for a mixing ratio of 1.0:1.0 at 180 min of bender element testing is equal to 361 m/s, while that of 0.8:1.0 is equal to 317 m/s. Hence, the mixing ratio of 1.0:1.0 is considered to be optimum, according to its maximum V_s_.

**Figure 13:**
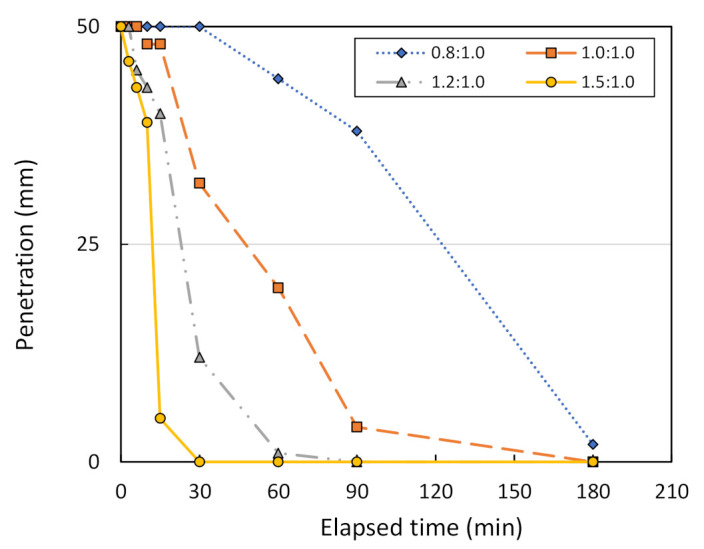
Penetration vs. the elapsed time of Vicat needle testing for metakaolin treated at 800 °C, using different mixing ratios.

**Figure 14:**
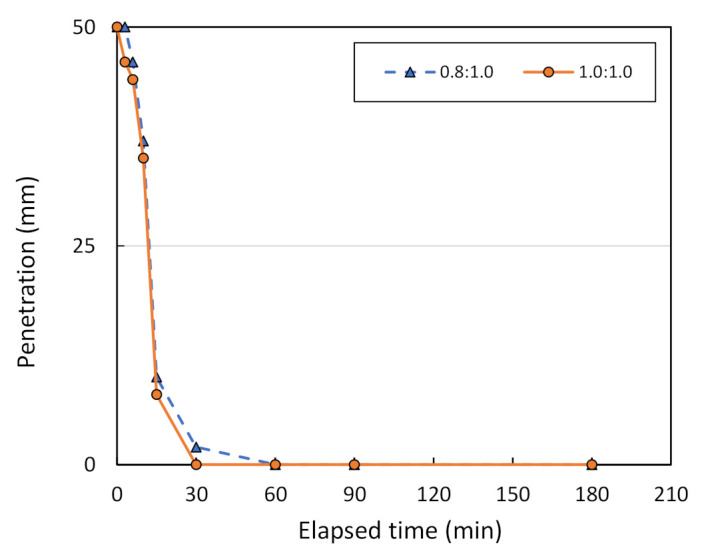
Penetration vs. the elapsed time of Vicat needle testing for metakaolin treated at 1000 °C, using different mixing ratios.

Figure 13 shows the Vicat needle test results on metakaolin geopolymer pastes with various mixing ratios, between metakaolin treated at 800 °C and the geopolymer solution. Note that the solution ratio between NaOH and Na_2_SiO_3_ is assumed to be 2.0:1.0. The initial setting time of metakaolin geopolymer pastes at 25-mm penetration is equal to 123, 48, 23, and 12 min for mixing ratios of 0.8:1.0, 1.0:1.0, 1.2:1.0, and 1.5:1.0, respectively. However, the final setting time at which there is no penetration is equal to 185, 180, 90, and 30 min for the mixing ratios of 0.8:1.0, 1.0:1.0, 1.2:1.0, and 1.5:1.0, respectively. Figure 14 shows the Vicat needle test results on metakaolin geopolymer pastes with various mixing ratios, between metakaolin treated at 1000 °C and a geopolymer solution. The initial setting time is equal to 12 min for the mixing ratio of 0.8:1.0, and 1.0:1.0. However, the final setting time is equal to 60 and 30 min for mixing ratios of 0.8:1.0 and 1.0:1.0, respectively. Hence, this finding confirms that a mixing ratio between the metakaolin powder and the geopolymer solution of 1.0:1.0 can be considered optimum for metakaolin treated at 1000 °C.

### 3.4. Relationship between Shear Wave Velocity and Setting Times

**Figure 15:**
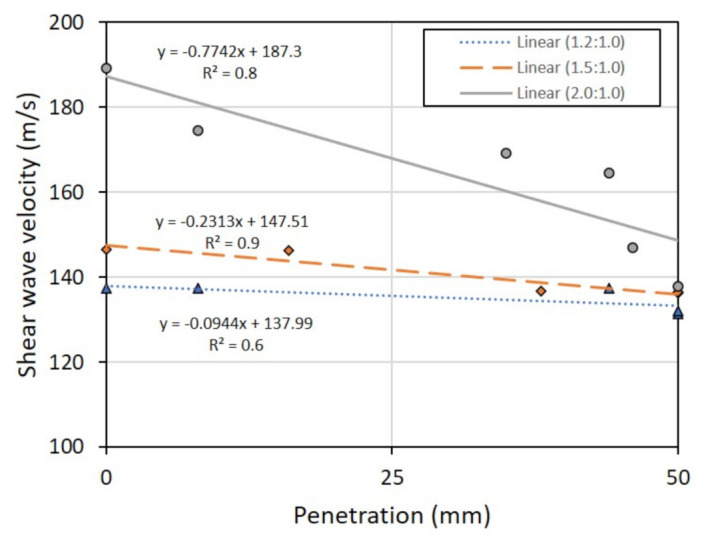
Shear wave velocity from bender element tests vs. penetration from Vicat tests.

Figure 15 shows the relationship between the shear wave velocity, V_s_, established via the bender element tests and the penetration through metakaolin geopolymer pastes established via the Vicat tests. For comparison purposes, this study assumes the metakaolin treated at 1000 °C, and the mixing ratio between the metakaolin powder and the geopolymer solution, to be 1.0:1.0. By varying the solution ratio between NaOH and Na_2_SiO_3_ from 1.2:1.0 to 2.0:1.0, the relationship between V_s_ and penetration appears to be linear. The initial setting time, set at 25-mm penetration, can be calculated as equal to 136, 142, and 168 m/s for solution ratios of 1.2:1.0, 1.5:1.0, and 2.0:1.0, respectively. Moreover, the final setting time when there is no penetration can also be calculated as equal to 138, 148, and 187 m/s for solution ratios of 1.2:1.0, 1.5:1.0, and 2.0:1.0, respectively. From these results, it can be established that the V_s_ of metakaolin geopolymer pastes reaches the maximum value when the solution ratio between NaOH and Na_2_SiO_3_ is equal to 2.0:1.0. Hence, a solution ratio of 2.0:1.0 is considered to be optimum in this study.

### 3.5. Effect of CBR on Mixing Methods for Laterite

**Figure 16:**
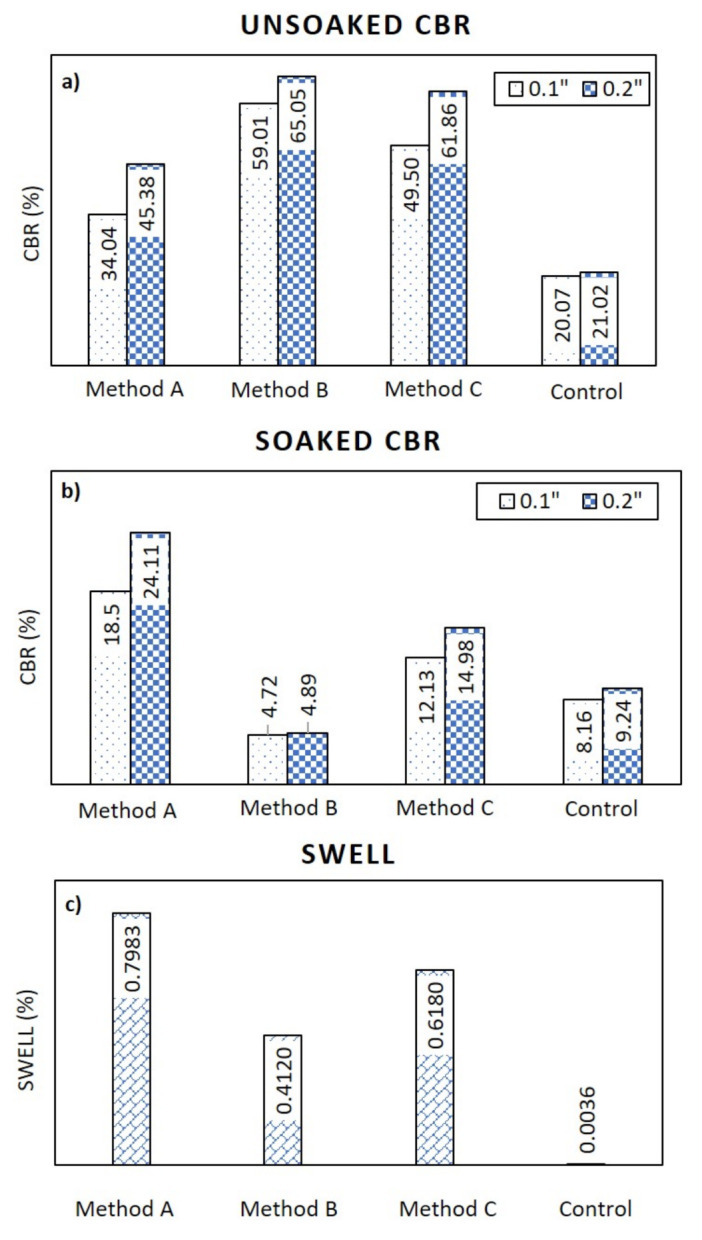
CBR test results for different mixing methods and conditions: (**a**) unsoaked (**b**) soaked (**c**) swelling.

Figure 16 shows the CBR test results on laterite soils mixed with metakaolin geopolymer. For comparison, this study assumes the solution ratio between NaOH and Na_2_SiO_3_ to be 2.0:1.0, and the mixing ratio between the metakaolin treated at 1000 °C and the geopolymer solution to be 1.0:1.0. Figure 16a shows the unsoaked CBR test results. The control laterite soil sample, which was compacted at an optimum moisture content of 10.17%, showed unsoaked CBR test results of 20.07% at 0.1 inches of penetration, as well as 21.02% at 0.2 inches of penetration. Mixing method A showed unsoaked CBR test results of 34.04% at 0.1 inches, as well as 45.38% at 0.2 inches of penetration. Moreover, mixing method B yielded the maximum unsoaked CBR test results of 59.01% at 0.1 inches, as well as 65.05% at 0.2 inches of penetration. Finally, mixing method C presented unsoaked CBR test results of 49.50% at 0.1 inches, as well as 61.86% at 0.2 inches of penetration. From these results, mixing method B is considered to show the best performance in the unsoaked CBR tests, and its CBR values were about 3 times higher than those of the control sample. Figure 16b shows the soaked CBR test results. The control laterite soil sample, which was compacted at an optimum moisture content of 10.17%, showed soaked CBR test results of 8.16% at 0.1 inches, as well as 9.24% at 0.2 inches of penetration. It is clear that mixing method A gave the maximum soaked CBR test results of 18.50% at 0.1 inches, as well as 24.11% at 0.2 inches of penetration. Moreover, its CBR values were about 3 times higher than those of the control sample. Figure 16c shows the swelling CBR test results. The control laterite sample, which was compacted at the optimum moisture content of 10.17%, showed swelling CBR test results of 0.0036%. It can be observed that method B provides the best swelling CBR test results of 0.41%.

## 4. Conclusions

This study uses piezoelectric ceramic sensors in combination with bender element tests to determine the initial and final setting times of metakaolin geopolymer pastes. These setting times are investigated via the shear wave velocity from the bender element and are compared to the penetration tests using the Vicat needle apparatus. Metakaolin was prepared from kaolin, with thermal treatment at 0 (control sample), 200, 800, 1000, and 1200 °C. These metakaolin samples were then mixed with the geopolymer solution in various ratios. Moreover, the geopolymer solution was prepared by adding 10 normal concentrations of sodium hydroxide (10 N NaOH) to sodium silicate (Na_2_SiO_3_) at various ratios. From the findings of this study, it can be summarized that:

- By varying the treatment temperature, the solution ratio between sodium hydroxide (NaOH) and sodium silicate (Na_2_SiO_3_), as well as the mixing ratio between the metakaolin powder and the geopolymer solution, their optimum value can be investigated. In other words, the optimum treatment temperature in producing metakaolin is herein found to be 1000 °C; the optimum solution ratio and the optimum mixing ratio were found to be 2.0:1.0 and 1.0:1.0, respectively.
- Assuming solution ratios between NaOH and Na_2_SiO_3_ to be 1.2:1.0, 1.5:1.0, and 2.0:1.0, the relationships between shear wave velocity and Vicat penetration appear to be linear, for a mixing ratio between the metakaolin treated at 1000 °C and the geopolymer solution of 1.0:1.0. According to these three solution ratios, the initial setting time was found to be 136, 142, and 168 m/s, respectively, but the final setting time was found to be 138, 148, and 187 m/s, respectively. Moreover, the optimum solution ratio was found to be 2.0:1.0, according to the maximum shear wave velocity.
- Assuming the solution ratio between NaOH and Na_2_SiO_3_ to be 2.0:1.0, as well as assuming the mixing ratio between metakaolin treated at 1000 °C and the geopolymer solution to be 1.0:1.0, CBR tests can be conducted on laterite soils mixed with metakaolin geopolymer and on control laterite soil samples. From the tests, mixing method B is found to be the best for the unsoaked CBR test; its CBR values were about 3 times higher than the control sample. For the soaked CBR test, mixing method A was found to be the best because its CBR values were about 3 times higher than the control sample. However, mixing method B was found to be the best for the swelling CBR tests as it demonstrated the best CBR test results.
- For further study, there are two recommendations. First, a study that extensively focuses on the strength development of metakaolin geopolymer pastes should be conducted, although the current study focuses on the shear wave velocity, which might imply their strength. Second, the use of scanning electron microscopy (SEM) testing was also recommended, in order to further observe the microstructure of metakaolin geopolymer pastes.

## Figures and Tables

**Table 1:** Chemical compositions of kaolin.

Chemical Composition	Content (%)
SiO_2_	65–70
Al_2_O_3_	17–20
K_2_O	4.20
Na_2_O	3.30
MgO	1.66
Fe_2_O_3_	˂1.60
TiO_2_	0.10
CaO	0.07

**Table 2:** Basic engineering properties of kaolin.

Properties	Unit
Moisture	1.0% max.
Particle sizes that pass through mesh No. 400 (38 μm)	100%
Finer than 2 microns	30–40%
pH	6.5 ± 1
Oil absorption	37 ± 2 gm/100 gm
Brightness Liquid limit Plastic limit Plastic index Total unit weight	80 ± 2% 46.84% 34.19% 12.65% 2.36 T/m^3^

**Table 3:** Basic engineering properties of the laterite soil samples.

Properties	Unit
Liquid Limit, LL	20%
Plastic Limit, PL	13.53%
Plastic Index, PI	6.47%
Specific gravity, G_s_	2.76
Maximum dry unit weight	2.069 T/m^3^
Optimum moisture content	10.17%
